# Changes in vitamin D and calcium metabolism markers in patients undergoing adjuvant chemotherapy for breast cancer

**DOI:** 10.1186/s12885-021-08563-4

**Published:** 2021-07-15

**Authors:** Marie Viala, Nelly Firmin, Célia Touraine, Stéphane Pouderoux, Manon Metge, Lobna Rifai, Gilles Romieu, Hélène de Forges, Lise Roca, Séverine Guiu, Véronique D’Hondt, William Jacot

**Affiliations:** 1 Department of Medical Oncology, Institut du Cancer de Montpellier (ICM), University of Montpellier, 208 avenue des Apothicaires, 34090 Montpellier, France; 2 INSERM U1194 – IRCM, Montpellier, France; 3 Biometrics Unit, Institut du Cancer de Montpellier (ICM), Univ. Montpellier, Montpellier, France; 4 Clinical Research Center, Institut du Cancer de Montpellier (ICM), Univ. Montpellier, Montpellier, France; 5 Department of Clinical Research and Innovation, Institut du Cancer de Montpellier (ICM), Univ. Montpellier, Montpellier, France; 6 Department of Clinical Research and Innovation, Centre Hospitalier Universitaire de Nîmes, Nîmes, France

**Keywords:** Hypercalciuria, Vitamin D deficiency, Adjuvant chemotherapy, Breast cancer

## Abstract

**Background:**

Changes in calcium metabolism and calcium urinary excretion during chemotherapy have not been thoroughly assessed in patients with early breast cancer (EBC), a population who frequently present vitamin D insufficiency. As hypercalciuria is a classical contra-indication to vitamin D (VD) supplementation, this study evaluated changes in VD and calcium metabolism parameters in patients with EBC undergoing adjuvant chemotherapy (CT).

**Methods:**

In patients with EBC who received six cycles of adjuvant CT, VD and calcium parameters were monitored at inclusion, and then every 3 weeks, at each CT cycle initiation. The primary endpoint was the percentage of patients showing hypercalciuria during adjuvant CT (between Day 1, Cycle 1 [D1C1] and Day 1, Cycle 6 [D1C6]).

**Results:**

The primary endpoint could be evaluated in 82 patients. Most patients (*n* = 66, 80.5%) had VD insufficiency (< 30 ng/mL) at baseline. Hypercalciuria was detected in 29 patients (35.4%; 95% CI: 25.6–46.5) between D1C1 and D1C6, but was not clinically significant in any of the affected patients. The percentage of hypercalciuria events was not different between patients with sufficient and insufficient baseline VD levels (34.8% vs. 37.5%), and between patients who received or not VD supplementation (37.5% vs. 34.5%,).

**Conclusions:**

This comprehensive study on VD and calcium parameter changes in patients with EBC during adjuvant chemotherapy shows that hypercalciuria is a frequent abnormality in this setting, although asymptomatic. Therefore, it should not be considered as a limitation for high dose VD supplementation in this population.

**Trial registration:**

EudraCT:2014-A01454-43. Registered 29 august 2016.

**Supplementary Information:**

The online version contains supplementary material available at 10.1186/s12885-021-08563-4.

## Background

Breast cancer remains a major public health issue with a prevalence of 1700,000 new cases per year worldwide, and 522,000 deaths in 2012 [1]. Most breast cancers are diagnosed at an early stage, with a 5-year survival rate of at least 99% for patients with localized tumors (tumor size < 2 cm and no nodal invasion), but only of 85% for patients with axillary nodal invasion. In early-stage breast cancer (EBC), breast-conserving surgery or mastectomy with lymph node evaluation (sentinel lymph node resection or axillary lymph node dissection) is the milestone of the treatment strategy [2]. The indication of adjuvant systemic treatments (chemotherapy and endocrine therapies) relies on the tumor clinical and pathological characteristics.

Standard chemotherapy is based on the combination of anthracycline and taxanes [3]. In patients with HER2-overexpressing tumors, trastuzumab is administered in addition to chemotherapy for 1 year [4]. Adjuvant chemotherapy, its complications (such as chemotherapy-induced menopause), and the often associated corticosteroids may lead to modifications of the phosphorus-calcium balance and bone metabolisms [5], and consequently to adverse events, such as osteoporosis and associated fractures [6]. Moreover, vitamin D insufficiency is frequently observed in patients with EBC [7, 8]. For instance, in a previous study, we reported that in a sample of 77 patients with EBC treated with neoadjuvant chemotherapy, 79.5% had baseline vitamin D insufficiency and 97.4% at treatment end [5]. Therefore, calcium and vitamin D supplements are indicated in this population. However, the recommended dosages (400–500 IU/day of vitamin D and 1 g of calcium) do not correct these deficiencies [9–11]. In agreement, Jacot and al suggested that higher doses of vitamin D and calcium are needed to improve vitamin D and calcium deficiency in patients with EBC ([12]). Moreover, no data is available on the changes in phosphorus and calcium metabolism parameters, including urine calcium concentration, in patients with EBC treated with adjuvant chemotherapy. Importantly, although multiple factors modulate urine calcium concentration, its increase is classically considered as a contra-indication for vitamin D supplementation, because it could indicate a biological overdose of this vitamin. Therefore, before testing higher vitamin D and calcium dosages in this population, it is important to monitor the modification of calcium and vitamin D parameters and calcium urinary excretion during chemotherapy, associated or not with VD supplementation, to better understand the consequences of adjuvant treatments on this metabolic pathway.

The aim of this prospective study was to analyze changes in vitamin D and calcium metabolism parameters during adjuvant chemotherapy in patients with EBC, and especially the changes in calcium urinary excretion (primary objective).

## Methods

### Study design

CALCIOBS was an open-label non-randomized monocentric observational study carried out in our comprehensive cancer center between December 2015 and May 2017 to evaluate vitamin D and calcium parameters in patients with EBC treated with adjuvant chemotherapy. This study was approved by the local ethics committee (CPP Mediterannée Sud) and the national review board (ANSM, number 2014A01454–43). All patients provided a written informed consent prior to participation in the study. The study was performed in accordance with the Good Clinical Practices Requirements and the Helsinki Declaration.

### Patients

Inclusion criteria were: ≥18-year-old patients with EBC, with Eastern Cooperative Oncology Group (ECOG) score of 0 or 1, and programmed to receive six cycles of adjuvant chemotherapy. The main exclusion criteria were: metastatic disease, another cancer treated in the previous 3 years, contra-indication to calcium or cholecalciferol administration (severe allergic reactions to vitamin D or calcium supplementation or to excipients, diseases or conditions that cause hypercalcemia and/or hypercalciuria, calcium nephrolithiasis, tissue calcification, hypervitaminosis D), significant comorbidities (e.g. uncontrolled endocrine disease, pre-existing disorder in the phosphorus-calcium balance in the last 3 years, osteopenia or osteoporosis requiring treatment with calcium and vitamin D treatment), and any concomitant treatment with experimental products.

### Endpoints and other assessments

The primary endpoint was the percentage of patients with hypercalciuria between the first day of the first adjuvant chemotherapy cycle (D1C1) and the first day of the last adjuvant chemotherapy cycle (D1C6), as reported by the clinician at the study end. The secondary endpoints were: (1) baseline vitamin D and calcium parameters, (2) percentage of patients with vitamin D normalization at D1C6 in the sub-population of patients with vitamin D insufficiency at baseline who received calcium and vitamin D supplementation (as reported by the clinician at the study end or as serum level ≥ 30 ng/mL at DIC6), (3) changes in vitamin D and calcium parameters between D1C1 and D1C6, (4) predictive value of baseline biomarkers on baseline vitamin D level, and (5) predictive value of baseline variables on hypercalciuria occurrence.

At inclusion, serum vitamin D levels and the following calcium parameters were evaluated: blood levels of calcium, phosphorus, albumin, urea, creatinine and parathyroid hormone (PTH), as well as 24-h urinary calcium excretion. These parameters were then assessed at day 1 of each new chemotherapy cycle. In patients with baseline vitamin D insufficiency, prescription of calcium and vitamin D supplementation at the currently recommended doses (1 g and 400–500 IU per day, respectively) was left to the investigator’s discretion [9, 11, 13].

Vitamin D insufficiency was defined as a serum vitamin D level lower than 30 ng/mL (75 nmol/L). The thresholds of continuous variables were based on the median of the study population; the normal/abnormal status of biological variables with a validated range was based on the local standard cut-offs (calcium: 2.15–2.5 mmol/l; phosphorus: 0.81–1.45 mmol/l; PTH: 15–65 ng/L). Hypercalciuria was defined as a 24-h urinary calcium level > 7 mmol.

### Statistical analyses

All the analyses were done on the evaluable population, defined as the eligible patients who completed the six cycles of chemotherapy or who withdrawn from the study after the occurrence of hypercalciuria. Based on a hypercalciuria frequency of 50% [14], a sample size of 82 patients gave a precision expressed by a two-sided 95% confidence interval (CI) width of 21.6%. Descriptive statistics were performed in the whole populations and in the two groups (sufficient and insufficient vitamin D at baseline). Baseline characteristics were described as median and range (quantitative variables) and number of observations and percentages (qualitative variables). The number of missing values was reported and the percentages were calculated excluding missing values (unless otherwise specified). Quantitative variables were compared with the Kruskal–Wallis test by ranks, and qualitative variables with the chi-square or Fisher’s exact test (when *n* < 5). The 95% CI for the primary endpoint was calculated using a logit transform. The laboratory parameter changes were described using boxplots at each chemotherapy cycle in the whole population and in each group. Additional analyses were performed using linear mixed models with random intercept and slope coefficients to predict the mean trajectories of the biological parameters in each group and to test time-effects and trajectory differences between groups. Logistic regressions were used to determine which baseline laboratory parameters were associated with baseline vitamin D deficiency and which baseline clinical characteristics and laboratory parameters were associated with hypercalciuria occurrence. All tests were two-sided and *p*-values < 0.05 were considered as statistical significant. All analyses were performed using the Stata software (version 13.0).

## Results

### Baseline patients’ characteristics

Between November 2015 and May 2017, 95 patients were prospectively included in the study. Among them, 13 patients were excluded from the analysis due to consent withdrawal (*n* = 8), metastatic disease (*n* = 3), no fulfillment of the study procedures (*n* = 1), and toxicity leading to chemotherapy discontinuation (*n* = 1). Therefore, the primary endpoint could be evaluated in 82 patients. The median follow-up was 17.3 weeks (range: 15.3–23.1).

The patients’ median age was 53 years (range: 20–71) (Table 1). At baseline, 35 (43.2%) patients were pre-menopausal, and 46 (56.8%) post-menopausal (*n* = 1 missing data). Most tumors were classified as stage I or II (91.3%). Moreover, 72 tumors (87.8%) were hormone receptor-positive, 10 (12.3%) HER2-positive, and 7 (8.5%) were triple negative. Overall, 79 patients (96%) received anthracycline and taxane-based sequential chemotherapy of six cycles. Trastuzumab was administered in addition to chemotherapy to 11 patients (13.4%). The median treatment period was 15 weeks (range: 14.9–18.7).

**Table 1:** Patients‘demographic, clinical and histological characteristics. Patients were divided in two groups: vitamin D sufficient and insufficient using the baseline serum vitamin D level cut-off of 30 ng/mL (75 nmol/L)

	Vitamin D Sufficient (***n*** = 16)		Vitamin D Insufficient (***n*** = 66)		Total (***n*** = 82)		***p***-value
**DEMOGRAPHIC DATA**							
**Age** (years), median [range]	55	[20–68]	52.5	[35–71]	53	[20–71]	**0.6**
**Menopausal status**, n (%)							
Pre-menopausal	6	(37.5)	29	(44.6)	35	(43.2)	**0.6**
Post-menopausal	10	(62.5)	36	(55.4)	46	(56.8)	
*Missing*	*0*		*1*		*1*		
**CLINICAL DATA**							
**ECOG score**, n (%)							
0	16	(100)	59	(89.4)	75	(91.5)	**0.3**
1	0	(0)	7	(10.6)	7	(8.5)	
**Weight** (kg), median [range]	62	[48–88]	64.5	[40–95]	64	[40–95]	**0.7**
**Height** (cm), median [range]	164	[157–170]	161	[149–178]	162.5	[149–178]	**0.3**
**BSA (**m^**2**^**)**, median [range]	1.71	[1.46–1.93]	1.71	[1.32–2.06]	1.71	[1.32–2.06]	**0.9**
**BMI**							
< 25	10	(62.5)	36	(54.5)	46	(56.1)	**0.6**
≥ 25	6	(37.5)	30	(45.5)	36	(43.9)	
**HISTOLOGICAL DATA**							
**Histological grade**, n (%)							
1	0	(0)	2	(3.0)	2	(2.5)	**0.7**
2	8	(50)	39	(59.1)	47	(57.3)	
3	8	(50)	25	(37.9)	33	(40.2)	
**Perivascular invasion**, n (%)							
No	10	(62.5)	48	(72.7)	58	(70.7)	**0.5**
Yes	6	(37.5)	18	(27.3)	24	(29.3)	
**Estrogen receptor**, n (%)							
< 10%	1	(6.2)	9	(13.6)	10	(12.2)	**0.7**
≥ 10%	15	(93.8)	57	(86.4)	72	(87.8)	
**Progesterone receptor**, n (%)							
< 10%	3	(18.7)	18	(27.3)	21	(25.6)	**0.7**
≥ 10%	13	(81.3)	48	(72.7)	61	(74.4)	
**HER2 +**, n (%)							
No	14	(87.5)	57	(87.7)	71	(87.7)	**1**
Yes	2	(12.5)	8	(12.3)	10	(12.3)	
*Missing*	*0*		*1*		*1*		
**Triple negative**, n (%)							
No	15	(93.8)	60	(90.9)	75	(91.5)	**1**
Yes	1	(6.2)	6	(9.1)	7	(8.5)	
**IJCC 2016 staging**, n (%)							
I	9	(56.3)	21	(32.8)	30	(37.5)	**0.5**
IIA	5	(31.2)	21	(32.8)	26	(32.5)	
IIB	2	(12.5)	15	(23.4)	17	(21.3)	
IIIA	0	(0.0)	6	(9.4)	6	(7.5)	
IIIC	0	(0.0)	1	(1.6)	1	(1.2)	
*Missing*	*0*		*2*		*2*		

*BSA* Body Surface Area, *BMI* Body Mass Index, *SBR* Scarff-Bloom and Richardson, *IJCC* International Joint Committee on Cancer

The median baseline vitamin D concentration was 20.7 ng/mL (range: 2.9–55). Considering a threshold of 30 ng/mL, 66 (80.5%) patients presented vitamin D insufficiency (median vitamin D value: 19.2 ng/mL, range: 2.9–29.9), and 16 (19.5%) patients did not (median vitamin D value: 37.5 ng/mL, range: 30.2–55]. Moreover, nine (11%) patients received calcium and/or vitamin D supplementation: five received both, one only calcium, and three only vitamin D. The baseline status of the assessed laboratory parameters, including PTH, calcium and phosphorus, was not correlated with vitamin D baseline insufficiency (Tables 1 and 2).

**Table 2:** Baseline serum levels of vitamin D, calcium, phosphorus, albumin, urea, creatinine and parathyroid hormone (PTH)

	Vitamin D Sufficient (***n*** = 16)		Vitamin D Insufficient (***n*** = 66)		Total (***n*** = 82)		***p***-value
**Albumin (g/L)**, median [range]	45	[40–51]	44	[37–53]	44	[37–53]	0.2
*Missing*	*0*		*1*		*1*		
**PTH (ng/mL)**, median [range]	38	[17–61]	42	[13.5–124]	42	[13.5–124]	0.2
*Missing*	*2*		*3*		*5*		
**PTH**, n (%)							
Normal	14	(100)	55	(87.3)	69	(89.6)	0.3
Abnormal	0	(0)	8	(12.7)	8	(10.4)	
*Missing*	*2*		*3*		*5*		
**Calcium (mmol/L)**, median [range]	2.42	[2.25–2.84]	2.39	[2.15–2.56]	2.39	[2.15–2.84]	0.1
*Missing*	*0*		*2*		*2*		
**Calcium**, n (%)							
Normal	13	(81.3)	60	(93.7)	73	(91.3)	0.1
Abnormal	3	(18.7)	4	(6.3)	7	(8.7)	
*Missing*	*0*		*2*		*2*		
**Phosphorus (mmol/L)**, median [range]	1	[0.88–1.29]	1	[0.65–1.61]	1	[0.65–1.61]	0.7
*Missing*	*0*		*6*		*6*		
**Phosphorus (N)**							
Normal	16	(100)	53	(88.3)	69	(90.8)	0.3
Abnormal	0	(0)	7	(11.7)	7	(9.2)	
*Missing*	*0*		*6*		*6*		
**Urea (mmol/L)**, median [range]	4.5	[2.9–8]	4.7	[2.2–8.2]	4.7	[2.2–8.2]	0.9
*Missing*	*1*		*6*		*7*		
**Creatinine (μmol/L)**, median [range]	63	[50–102]	63	[37–91]	63	[37–102]	0.4
*Missing*	*0*		*1*		*1*		
**25 OH vit D (ng/mL)**, median [range]	37.5	[30.2–55]	19.2	[2.9–29.9]	20.65	[2.9–55]	**< 0.001**
*Missing*	*0*		*0*		*0*		

### Hypercalciuria occurrence during adjuvant chemotherapy: primary endpoint

At baseline, hypercalciuria was detected in 11 patients (15%). During adjuvant chemotherapy (between D1C1 and D1C6), hypercalciuria was observed in 29 of the 82 evaluable patients (35.4%) [95% CI: 25.6–46.5] (Table 3). Its frequency was not different in patients with and without baseline vitamin D sufficient concentration: 37.5% (95% CI: 16.1–65.2) versus 34.8%, (95% CI: 24.1–47.4) (*p =* 0.8) (Table 3). Similarly, hypercalciuria occurrence was not different in patients with and without calcium and/or vitamin D supplementation (37.5% [95% CI: 8.7–79.2] versus 34.5% [95% CI: 23.1–47.9], respectively; *p =* 0.7), and in pre-menopausal and post-menopausal patients (34.3% [95% CI: 20–52.1] versus 34.8% [95% CI: 22.1–50], respectively; *p* = 0.4) (Table 4).

**Table 3:** Occurrence of hypercalciuria during chemotherapy in patients with vitamin D sufficient and insufficient levels, using the baseline serum vitamin D level cut-off of 30 ng/mL (75 nmol/L)

	Sufficient (***N*** = 16)		Insufficient (***N*** = 66)		Total (***N*** = 82)		
	N	% [95% CI]	N	% [95% CI]	N	% [95% CI]	***p***-value
**Hypercalciuria**							
**No**	10	62.5 [34.8–83.9]	43	65.2 [52.6–75.9]	53	64.6 [53.5–74.4]	0.8
**Yes**	6	37.5 [16.1–65.2]	23	34.8 [24.1–47.4]	29	35.4 [25.6–46.5]

**Table 4:** Occurrence of hypercalciuria according to calcium and/or vitamin D supplementation (top) and baseline menopausal status (bottom)

**Vitamin D and/or calcium supplementation (*N*** **= 66)**					
	**Yes (*n*** **= 9)**		**No (*n*** **= 58)**		***p*-value**
**Hypercalciuria**					
**No**	5	62.5 [20.8–91.3]	38	65.5 [52.1–76.9]	0.7
**Yes**	3	37.5 [8.7–79.2]	20	34.5 [23.1–47.9]	
**Baseline Menopausal status (*N*** **= 81)**					
	**Yes (*n*** **= 46)**		**No (*n*** **= 35)**		
**Hypercalciuria**					
**No**	30	65.2 [50–77.9]	23	65.7 [47.9–80]	0.4
**Yes**	16	34.8 [22.1–50]	12	34.3 [20–52.1]	

**Fig. 1:**
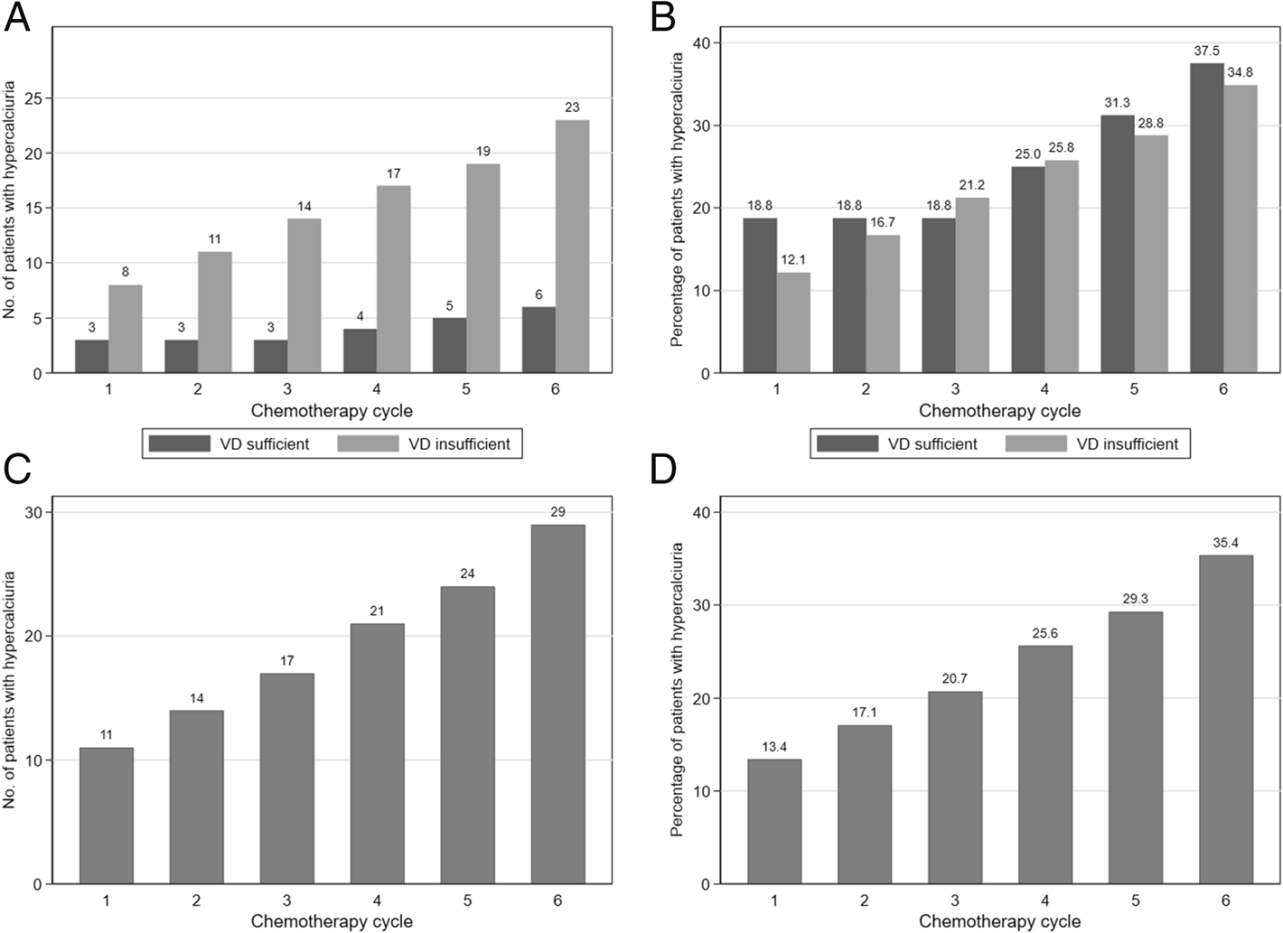
Cumulative number (**A** and **C**) and percentage (**B** and **D**) of patients with EBS with at least one occurrence of hypercalciuria during chemotherapy. In **A** and **B**, patients were divided in two groups (vitamin D sufficient, *n* = 16, and insufficient, *n* = 66) using the baseline serum vitamin D level cut-off of 30 ng/mL (75 nmol/L). **C** and **D** present the same data for the whole population (*n* = 82)

Hypercalciuria was more frequently detected in patients receiving docetaxel than paclitaxel (44% versus 21.4%, *p = 0.036*). No hypercalciuria was reported in patients treated with methotrexate-based chemotherapy (*n* = 3). Considering the 24-h urinary calcium level during adjuvant chemotherapy, the cumulative number of patients who developed hypercalciuria (24-h urinary calcium level > 7 mmol) increased linearly with the number of chemotherapy cycles (Fig. 1). Specifically, 13.4% of patients developed hypercalciuria during the first cycle and the cumulative rate at treatment end was 42% (Fig. 1).

### Blood parameters of the vitamin D – calcium metabolism

**Fig. 2:**
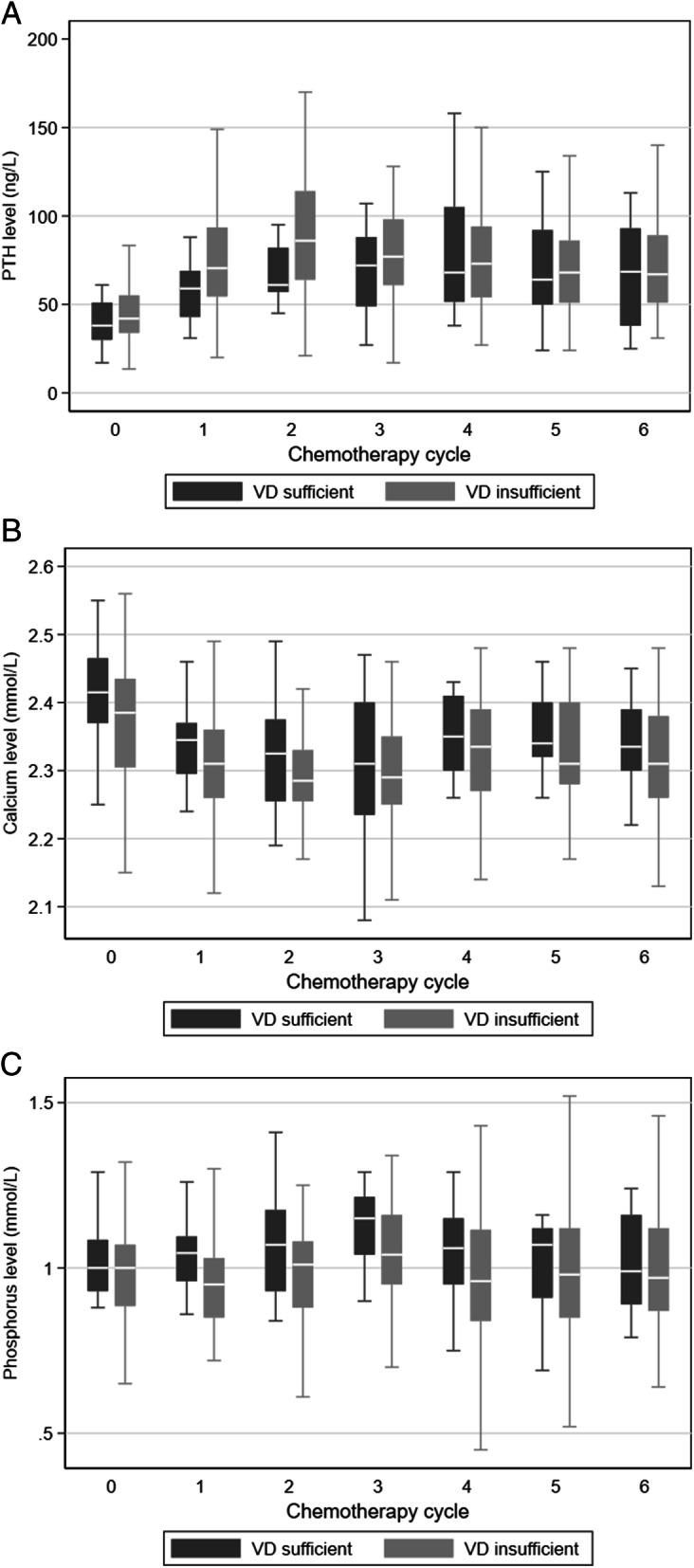
PTH (**A**), calcium (**B**) and phosphorus (**C**) levels changes during chemotherapy according to the serum baseline vitamin D concentration. Patients were divided in two groups (vitamin D sufficient, *n* = 16, and insufficient, *n* = 66) using the baseline serum vitamin D level cut-off of 30 ng/mL (75 nmol/L)

**Fig. 3:**
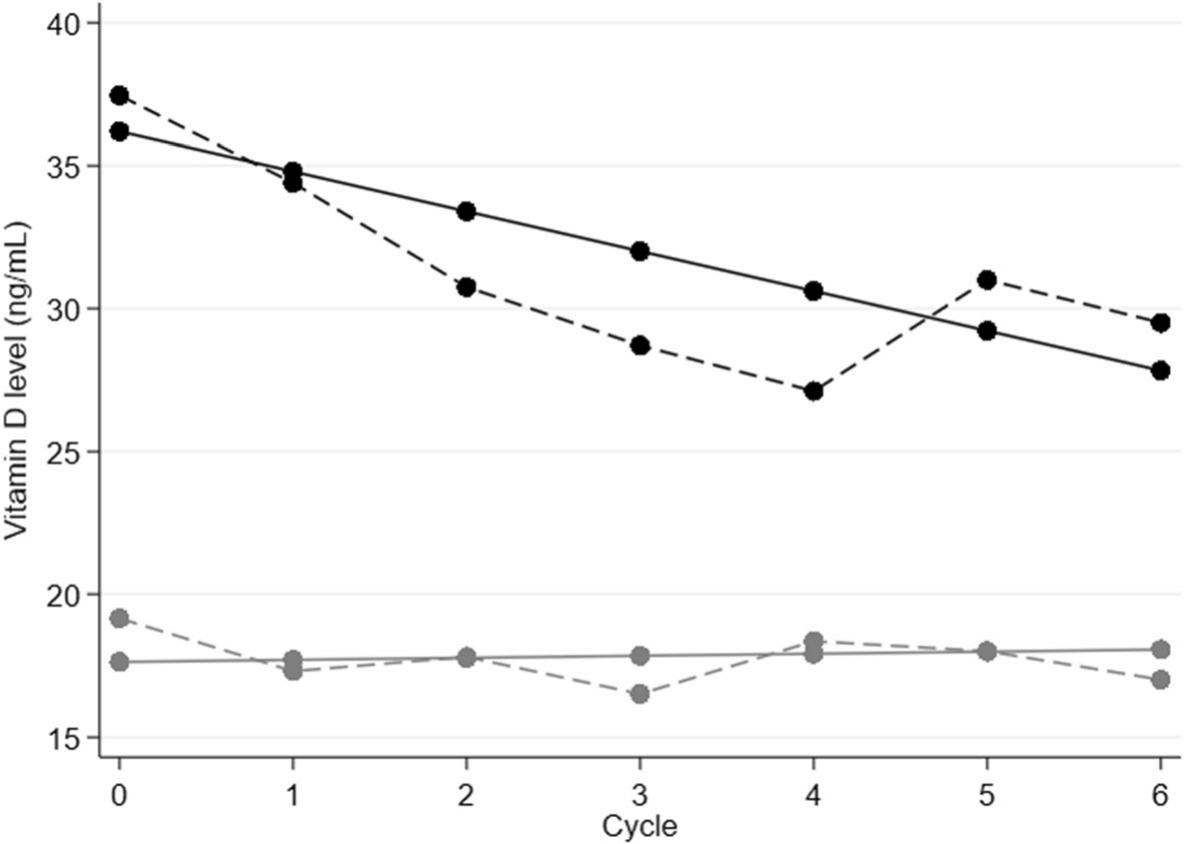
Predicted (solid lines) and observed (dashed lines) vitamin D serum concentration during chemotherapy. Patients were divided in two groups: vitamin D sufficient (*n* = 16, black lines) and insufficient (*n* = 66, grey lines) using the baseline serum vitamin D level cut-off of 30 ng/mL (75 nmol/L)

The serum levels of vitamin D, PTH, phosphorus and calcium, from D1C1 to D1C6, are presented in Fig. 2 and supplementary data. At treatment end, the median vitamin D levels were 20 ng/mL (versus 20.7 ng/mL at baseline) in the whole population, 29.5 ng/mL (range: 20–39) in the vitamin D sufficient group, and 17 ng/mL in the vitamin D insufficient group (range: 9–37.4). Although the vitamin D level was significantly different between groups during chemotherapy (*p* < 0.001 for each cycle), 50% of patients (7/16; *n* = 2 missing data) with baseline vitamin D sufficiency displayed vitamin D insufficiency at treatment end. The linear mixed model analysis found a significant decrease of the mean vitamin D level in the vitamin D sufficient group that was estimated at 1.4 ng/mL between cycles (slope estimate = − 1.4, *p* < 0.001) (Fig. 3). Among the 66 patients with insufficient vitamin D level at baseline, 5 corrected their vitamin D level with supplementation (7.8%, *n* = 2 missing data).

Serum PTH concentration was significantly different between the vitamin D sufficient and insufficient groups at C1 (*p* =0.044) and C2 (*p* =0.025). PTH level seemed to increase during chemotherapy compared with baseline (Fig. 2A). The linear mixed model analysis highlighted a significant increase of the mean PTH level during chemotherapy in both groups (estimated at 3.8 ng/mL between cycles, *p* < 0.001).

Serum calcium level during chemotherapy was lower in the vitamin D insufficient group than in the sufficient group (Fig. 2B), but this difference was not significant. Unlike PTH concentration, the mean calcium level significantly decreased (*p* < 0.02) during treatment (estimated at 0.01 mmol/L between cycles in the vitamin D sufficient group, but without significant difference compared with the vitamin D insufficient group).

Serum phosphorus concentration was lower in the vitamin D insufficient than sufficient group at C1 (*p* =0.018), but not at C2 and C3 (*p* < 0.10) (Fig. 2B). The linear mixed models analysis confirmed the difference in phosphorus concentration between groups (*p* =0.042), with an estimated mean phosphorus concentration difference of 0.07 mmol/L in the vitamin D insufficient vs. sufficient group.

### Baseline factors associated with baseline vitamin D insufficiency

No baseline laboratory parameter was significantly associated with baseline vitamin D insufficiency, although a trend towards a correlation between high calcium serum concentration and lower risk of baseline vitamin D insufficiency (Odds Ratio, OR = 0.003) was observed (*p* =0.051). None of the clinical parameters (e.g. weight, body mass index, age, and menopausal status) was significantly associated with baseline vitamin D insufficiency.

### Predictive value of baseline clinical-pathological variables on hypercalciuria occurrence during adjuvant chemotherapy

In univariate analysis, larger body surface area and lower albumin level at baseline were significantly correlated with hypercalciuria occurrence during adjuvant chemotherapy (body surface area ≥ 1.7 vs. < 1.7, OR = 2.68; *p* = 0.04 and serum albumin level ≥ 44 g/L vs. < 44 g/L, OR = 3.055; *p* = 0.023).

## Discussion

This prospective monocentric study evaluated the changes in blood and urinary vitamin D and calcium concentrations in patients with EBC during adjuvant chemotherapy. Our study confirmed the high frequency (80.5%) of baseline vitamin D insufficiency, in accordance with the literature. Crew et al. reported a 74% rate of vitamin D insufficiency before adjuvant chemotherapy in a cohort of 103 patients with EBC [7]. Similarly, our team found a baseline vitamin D insufficiency frequency of 79.4% in a previous cohort of patients with EBC receiving neoadjuvant chemotherapy, a rate that increased to 97.4% at treatment end. In this previous study, we also showed that vitamin D insufficiency was associated with bone metabolism imbalance: the levels of calcium and RANK ligand (RANKL) (a major stimulating factor of osteoclast formation and survival) decreased during chemotherapy, and those of osteoprotegerin (OPG), a protein that inhibits osteoclast recruitment, increased. The consequent decrease of the RANKL/OPG ratio suggests a dysregulation of a functional regulatory mechanism of bone turn-over [5]. In the present study, we confirmed, in the adjuvant setting, the significant decrease of serum vitamin D levels during chemotherapy, especially in the group with baseline vitamin D sufficiency. Indeed, 50% of patients in this group had vitamin D deficiency at the study end. Overall, 84.6% of the whole population presented vitamin D insufficiency at the study end (Supplementary Table 1). Thus, vitamin D insufficiency was consistently and frequently associated with breast cancer diagnosis and increased (frequency and severity) during chemotherapy treatment. However, baseline vitamin D insufficiency was not associated with any of the baseline clinical and laboratory variables. This vitamin D insufficiency may strongly increase the risk of skeletal morbidity and other calcium-related diseases.

The recommended daily calcium and vitamin D supplementation (200 to 400 IU of vitamin D per day and 1200 mg of calcium per day) is not enough, in most cases, to normalize the serum vitamin D levels during chemotherapy [15, 16]. Our team previously reported, in a prospective randomized trial, a 12% rate of vitamin D level normalization with the classical daily supplementation, which is comparable to the 7.8% rate observed in the present study. Altogether, these data indicate that vitamin D insufficiency in this population requires higher vitamin D and calcium doses to increase the rate of vitamin D level normalization to 30%, as shown in our previous study [12]. Previous studies reported an association between low baseline vitamin D levels and poor response to neoadjuvant chemotherapy. The retrospective study by Chiba et al. showed that baseline vitamin D deficiency increases by 2.65 times the risk of not reaching a pathological complete response after neoadjuvant chemotherapy [17]. This link between vitamin D levels and response to treatment [18] might strengthen the clinical interest to achieve sufficient vitamin D levels in patients with EBC during treatment. However, due to the multiple interactions of vitamin D with breast cancer outcome, kidney cancer clearance and the potential side effects of hypervitaminosis D, a better understanding of calcium metabolism is needed before increasing vitamin D supplementation in clinical practice. Indeed, the safety of higher vitamin D doses must be evaluated as required by the Health Regulatory Services and Ethics Committees, especially concerning hypercalciuric events, a potential side effect of vitamin D supplementation. Therefore, here, we evaluated changes in urine calcium levels in patients taking or not vitamin D supplementation during EBC treatment. Urinary calcium excretion is an important biomarker for understanding the phosphorus-calcium metabolism, and the 24-h urinary calcium excretion is routinely used to guide mineral supplementation in Europe [19]. However, there was no data on this parameter during chemotherapy, thus limiting the generalization of high dose vitamin D supplementation in these patients. In the present study, we detected baseline hypercalciuria in 15% of patients, in agreement with data in the general population (10%) [20]. Our results highlighted that hypercalciuria is frequent during chemotherapy (35%), but always clinically asymptomatic. The absence of symptoms might be due to the fact that urine calcium elevation in our patients was generally moderate, and clinical symptoms might appear only with chronic hypercalciuria (i.e. years) or with extreme concentrations. Moreover, hypercalciuria has never been described in the natural history of breast cancer. We found that hypercalciuria occurrence was not correlated with baseline vitamin D levels and with calcium and vitamin D supplementation, in accordance with the results by Gallagher et al. [21]. In patients with EBC undergoing adjuvant chemotherapy, hypercalciuria might be explained by several factors in addition to chemotherapy. In a previous study, we showed that dysregulation of the osteoclast/osteoblasts balance [5] occurs concomitantly with the decrease of serum vitamin D levels. Also, chemotherapy can sometimes induce chemical menopause due to ovarian failure (in pre-menopausal women). Moreover, it has been shown that post-menopausal women develop hypercalciuria more frequently because of the menopause-induced changes in calcium balance [22], resulting in bone loss [23]. In our study, almost half of patients were pre-menopausal at baseline. Hypercalciuria occurred independently of the menopausal status; nevertheless, most pre-menopausal patients might have developed at least transient ovarian failure due to chemotherapy, which could have modified bone metabolism. Thus, long-term evaluation of bone mineral density in these patients would allow testing a possible correlation between low bone density and hypercalciuria.

Transient hypercalciuria also has been reported in association with concomitant treatments, such as corticosteroids that are often used in combination with chemotherapeutic agents (anthracyclines and taxanes) as anti-emetic and anti-allergic drugs [24]. Hypercalciuria is due to the decrease of calcium absorption by the digestive tube, and an increase in its renal excretion, especially in patients receiving methylprednisolone doses higher than 20 mg per day. Calcium urinary level was not assessed at 6 months after chemotherapy completion in our study. Almost all patients received sequential chemotherapy (anthracyclines and taxanes), associated with systematic corticosteroids. Nevertheless hypercalciuria occurred more frequently in patients receiving docetaxel (44%) than in those receiving paclitaxel (21.4%), which might be explained by a higher cumulated dose of corticosteroids during the course of their treatment than in the paclitaxel group.

Finally, dietary changes, including those associated with chemotherapy, have been reported to induce hypercalciuria [22–26]. One limitation of the present study is the lack of dietary data from our patients during the study.

Although hypercalciuria had no clinical impact in our patients, it remains a classical contra-indication to vitamin D supplementation, and therefore the identification of baseline variables associated with hypercalciuria occurrence could allow the selection of patients with low hypercalciuria risk for high dose vitamin D supplementation. However, in our study, only the baseline body surface and serum albumin level were predictive of hypercalciuria occurrence. Data are scarce on predictive factors of hypercalciuria. Carvalho et al. found no correlation between body mass index and hypercalciuria in a cohort of post-menopausal women with osteoporosis [23], and Mente et al. showed a correlation between hypercalciuria and lower body mass index in a population of patients with kidney stone disease and hypercalciuria, as we previously described in our cohort of patients with breast cancer [18]. Thus, asymptomatic hypercalciuria appears to be a frequent, rarely predictable, and benign condition during adjuvant chemotherapy for EBV, and should not be a limitation to adequate vitamin D supplementation in this setting.

## Conclusions

In this prospective study of vitamin D level and calcium metabolism changes in patients with EBC during adjuvant chemotherapy, hypercalciuria occurred in 35% of patients, but was always asymptomatic, independently of the vitamin D level and of calcium and vitamin D supplementation. Standard supplementation was safe but ineffective in our population. Urinary calcium levels does not seem relevant in patients with EBC receiving adjuvant chemotherapy, and should not be considered as an exclusion criterion for vitamin D insufficiency correction, considering the multiple negative effects of such deficiency.

## Supplementary Information

**Additional file 1: Figure S1.** Vitamin D serum level changes during the six chemotherapy cycles in patients with baseline vitamin D sufficient (*n* = 16) and insufficient concentration (*n* = 66). **Table S1.** Vitamin D level changes during the six cycles of chemotherapy in patients with baseline sufficient and insufficient vitamin D concentration.

## Data Availability

The data that support the findings of this study are available from the authors upon reasonable request.

## Abbreviations

VD: Vitamin D
CT: Chemotherapy
D1C1: Day 1, Cycle 1
D1C6: Day 1, Cycle 6
EBC: Early Breast Cancer
HER2: Human EGF Receptor 2
RANKL: RANK ligand
OPG: Osteoprotegerin
ECOG: Eastern cooperative oncology group
Min: Minimum
Max: Maximum
PTH: Parathormone
IU: International unit
OR: Odds ratio
