# Supplementary material for: Changes in vitamin D and calcium metabolism markers in patients undergoing adjuvant chemotherapy for breast cancer
Source: BMC Cancer. 2021 Jul 15;21:815. doi: 10.1186/s12885-021-08563-4 (PMC8283899; doi:10.1186/s12885-021-08563-4)
Supplement: Supplementary file 1 — Additional file 1: Figure S1. Vitamin D serum level changes during the six chemotherapy cycles in patients with baseline vitamin D sufficient (n = 16) and insufficient concentration (n = 66). Table S1. Vitamin D level changes during the six cycles of chemotherapy in patients with baseline sufficient and insufficient vitamin D concentration. [file 12885_2021_8563_MOESM1_ESM.docx]

**Changes in vitamin D and calcium metabolism markers in patients undergoing
adjuvant chemotherapy for breast cancer**

Marie Viala^1^*, Nelly Firmin^1, 2^, Célia Touraine^3^, Stéphane Pouderoux^1^, Manon Metge^4^, Lobna Rifai^5^, Gilles Romieu^1^, Lise Roca^3^, Séverine Guiu^1,2^, Véronique D'Hondt^1, 2^, William Jacot^1, 2^

^1^Department of Medical Oncology, Institut du Cancer de Montpellier (ICM), University of Montpellier, France;

^2^INSERM U1194 – IRCM, Montpellier, France;

^3^Biometrics Unit, Institut du Cancer de Montpellier (ICM), Univ. Montpellier, Montpellier, France;

^4^Clinical Research Center, Institut du Cancer de Montpellier (ICM), Univ. Montpellier, Montpellier, France;

^5^Department of Clinical Research and Innovation, Institut du Cancer de Montpellier (ICM), Univ. Montpellier, Montpellier, France.

***Corresponding author:**

Dr Marie Viala
Institut du Cancer de Montpellier (ICM)
208 avenue des Apothicaires
34298 Montpellier
[marie.viala@icm.unicancer.fr](mailto:marie.viala@icm.unicancer.fr)
Tel: +33 4 67 61 47 04

Fax : +33 4 67 61 23 47

**Supplementary data:**

**Figure S1:** Vitamin D serum level changes during the six chemotherapy cycles in patients with baseline vitamin D sufficient (n=16) and insufficient concentration (n=66).

**
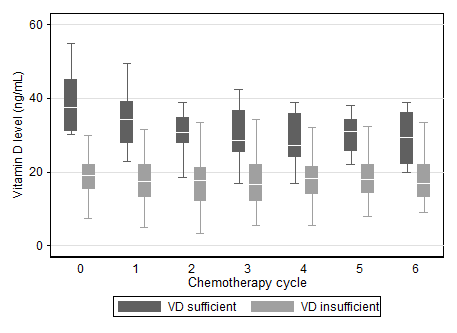
**

**Table S1**: Vitamin D level changes during the six cycles of chemotherapy in patients with baseline sufficient and insufficient vitamin D concentration

|  | **Baseline vit. D sufficient** | |  | **Baseline vit. D insufficient** | |  | **Total** | |
| --- | --- | --- | --- | --- | --- | --- | --- | --- |
|  | **N=16** | **%** |  | **N=66** | **%** |  | **N=82** | **%** |
| **Vit D insufficiency** |  |  |  |  |  |  |  |  |
| **Baseline** |  |  |  |  |  |  |  |  |
| Insufficient | 0 | 0 |  | 66 | 100 |  | 66 | 80.5 |
| **C1** |  |  |  |  |  |  |  |  |
| Insufficient | 4 | 25.0 |  | 62 | 96.9 |  | 66 | 82.5 |
| *Missing* |  |  |  | *2* |  |  |  |  |
| **C2** |  |  |  |  |  |  |  |  |
| Insufficient | 7 | 43.8 |  | 58 | 96.7 |  | 65 | 85.5 |
| *Missing* |  |  |  | *6* |  |  | *6* |  |
| **C3** |  |  |  |  |  |  |  |  |
| Insufficient | 8 | 50.0 |  | 61 | 95.3 |  | 69 | 86.3 |
| *Missing* |  |  |  | *2* |  |  | *2* |  |
| **C4** |  |  |  |  |  |  |  |  |
| Insufficient | 10 | 66.7 |  | 57 | 93.4 |  | 67 | 88.2 |
| *Missing* | *1* |  |  | *5* |  |  | *6* |  |
| **C5** |  |  |  |  |  |  |  |  |
| Insufficient | 6 | 37.5 |  | 60 | 93.8 |  | 66 | 82.5 |
| *Missing* |  |  |  | *2* |  |  | *2* |  |
| **C6** |  |  |  |  |  |  |  |  |
| Insufficient | 7 | 50.0 |  | 60 | 93.8 |  | 67 | 85.9 |
| *Missing* | 2 |  |  | 2 |  |  | 4 |  |
